# Isatidis Folium Represses Dextran Sulfate Sodium-Induced Colitis and Suppresses the Inflammatory Response by Inhibiting Inflammasome Activation

**DOI:** 10.3390/nu16193323

**Published:** 2024-09-30

**Authors:** You Chul Chung, Ami Lee, Chan Ho Jang, Jin Ah Ryuk, Hyunil Ha, Youn-Hwan Hwang

**Affiliations:** 1 KM Convergence Research Division, Korea Institution of Oriental Medicine, 1672 Yuseong-daero, Yuseong-gu, Daejeon 34054, Republic of Korea; jyc8385@kiom.re.kr (Y.C.C.); dmb01367@kiom.re.kr (A.L.); chjang78@kiom.re.kr (C.H.J.); yukjinah@kiom.re.kr (J.A.R.); hyunil74@kiom.re.kr (H.H.); 2 Korean Convergence Medical Science Major, KIOM School, University of Science & Technology (UST), Daejeon 34054, Republic of Korea

**Keywords:** inflammasome, ulcerative colitis, Isatidis Folium, anti-inflammatory agents, macrophages, caspase-1

## Abstract

Background/Objectives: Isatidis Folium (IF) has been used in traditional medicine for various ailments, and recent research highlights its anti-inflammatory, antiviral, and detoxifying properties. This study investigated the anti-inflammatory effects of a hydroethanolic extract of IF (EIF) on inflammasomes and colitis. Methods: Dextran sulfate sodium (DSS)-induced colitis model C57BL/6 mice were treated with DSS, mesalamine, or EIF (200 mg/kg). Parameters such as daily disease activity index (DAI), spleen weight, colon length, and histopathology were evaluated. Intestinal fibrosis, mucin, and tight junction proteins were assessed using Masson’s trichrome, periodic acid–Schiff, and immunohistochemistry staining. RAW264.7 and J774a.1 macrophages were treated with EIF and lipopolysaccharide, with cell viability assessed via the cell counting kit-8 assay, nitric oxide (NO) production with Griess reagent, and cytokine levels with the enzyme-linked immunosorbent assay. NF-κB inhibition was analyzed using the luciferase assay, and phytochemical analysis was performed using UPLC-MS/MS. Results: EIF mitigated weight loss, reduced DAI scores, prevented colon shortening, and attenuated mucosal damage, fibrosis, and goblet cell loss while enhancing the tight junction protein occludin. The anti-inflammatory effects of EIF in RAW264.7 cells included reduced NO production, pro-inflammatory cytokines, and NF-κB activity, along with inhibition of NLRP3 inflammasome responses in J774a.1 cells. The key constituents identified were tryptanthrin, indigo, and indirubin. Conclusions: Animal studies demonstrated the efficacy of EIF in alleviating colitis, suggesting its potential for treating inflammatory diseases.

## 1. Introduction

Inflammatory bowel disease (IBD) is a chronic condition characterized by inflammation of the intestines, with Crohn’s disease and ulcerative colitis being the main types. Symptoms typically include abdominal pain, diarrhea, bloody stools, and weight loss over several months [1,2]. Its pathogenesis is not yet clearly understood; however, it is believed to involve various environmental factors that weaken the immune response of the intestinal mucosa [3,4,5,6]. The intestinal barrier, composed of intestinal epithelial cells, protects the mucosal tissue from gut microbiota present in the intestinal lumen. In healthy individuals, non-pathogenic commensal bacteria coexist in the intestine without eliciting harmful immune responses, maintaining immune tolerance. However, when the barrier function is compromised, microorganisms can invade the mucosa and interact with immune cells via pattern-recognition receptors (PRRs), such as NOD-like receptors (NLRs) and Toll-like receptors (TLRs). Activation of these PRRs accelerates the immune response by promoting cytokine expression, including interleukin (IL)-1β and IL-18, ultimately leading to IBD development [4,7]. NOD-like receptor protein 3 (NLRP3) is a key regulator of gut homeostasis, with implications in Crohn’s disease. NLRP3, as a component of the inflammasome, induces the production of the inflammatory cytokines IL-1β and IL-18. Therefore, it is highly likely that NLRP3 plays a substantial role in IBD modulation [8,9,10].

The immune defense system of higher animals against pathogen infection is broadly divided into innate immunity and adaptive immunity. Innate immunity, serving as the first line of defense during early infection, is primarily executed by phagocytes, such as macrophages or dendritic cells [11]. PRRs on the cell membranes of these cells recognize various molecular patterns of pathogens, activating intracellular signaling systems and ultimately promoting the generation and secretion of pro-inflammatory cytokines [12,13]. TLRs are representative PRRs on the cell membrane; however, NLRs have been identified in the cytoplasm, performing functions similar to those of PRRs [14]. Among NLRs, NLRP3 is the most extensively studied receptor, activated by pathogen-associated molecular patterns, such as lipopolysaccharide (LPS), and damage-associated molecular patterns, such as ATP and uric acid crystals. Activated NLRP3 forms a protein complex called the inflammasome, cleaving pro-caspase-1 into active caspase-1. This active caspase-1 subsequently cleaves pro-IL-1β and pro-IL-18, increased by TLR/NF-κB signaling, into cleaved-IL-1β and -18, respectively, leading to their secretion outside the cell as active cytokines [15,16,17].

Recently, there has been active research on applying traditional herbal remedies used in Chinese medicine to IBD [18]. For example, *Hericium erinaceus* has shown the potential to exert beneficial effects in the treatment of IBD by reducing TNF and COX-2 levels and by increasing IL-10 levels in an ex vivo model composed of inflamed tissues obtained from patients with Crohn’s disease or ulcerative colitis [19,20].

Isatidis Folium (IF), the dried leaf of *Isatis indigotica* L., has a long history of use in traditional Chinese medicine as a herbal remedy for alleviating various symptoms. Historical records indicate its use since ancient times, with mentions of honey-soaked leaves being used to treat aphthous ulcers during the Song Dynasty and IF juice being noted for detoxifying medicines and poisons during the Weijin Dynasty. The “Ben Cao Zheng” records indicate that IF juice treated fever, swelling, and pain, whereas the “Ben Cao Gang Mu” from the Ming Dynasty suggests its effectiveness in detoxification, anti-pyresis, and anti-inflammation [21]. Owing to these medicinal properties, recent research has focused on verifying the pharmacological effects of IF, revealing its anti-inflammatory [22], antiviral [23], antiendotoxin [24], anti-wrinkle [25], and anti-atopic [26] properties. However, studies on its anti-inflammatory effects in relation to inflammasomes and the alleviation of colitis by IF are lacking. Thus, our study aimed to investigate the anti-inflammatory effects of IF in relation to inflammasomes and its alleviation of colitis using a hydroethanolic extract of IF (EIF).

## 2. Materials and Methods

### 2.1. Preparation of EIF

The extract of Isatis indigotica Fort. (IF) from Gwangmyeongdang Pharm (#FHM-K-17), located in Ulsan, Republic of Korea, was obtained using a detailed extraction process involving 70% ethanol as the solvent. The plant material underwent reflux extraction for 3 h. Following extraction, the solution was filtered to remove any residual plant debris, ensuring a pure extract. The filtered extract was then lyophilized, or freeze-dried, resulting in a fine EIF powder (product code: #KE-FHM-K-17) that was stable for further experimentation. This EIF powder was carefully stored at −20 °C to preserve its chemical integrity [27]. Phytochemicals, such as tryptanthrin (PubChem CID 73549), a natural indole quinazoline alkaloid in the form of a golden yellow powder with a molecular weight of 248.24 g/mol; indigo (PubChem CID 10215), a natural compound in the form of a dark blue crystalline powder with a molecular weight of 264.27 g/mol; and indirubin (PubChem CID 10177), a stable red isomer of blue indigo with a molecular weight of 262.26 g/mol, were identified in EIF by comparing retention times and mass spectral data (Table S1) using reference standards or as described by Liau et al., 2007 [28].

### 2.2. Animal Studies

#### 2.2.1. Animals

Animal studies, conducted under permit number 23-055 from the Institutional Animal Care and Use Committee of the Korea Institute of Oriental Medicine, followed the US National Institute of Health’s “Guide for the Care and Use of Laboratory Animals”. Seven-week-old male C57BL/6 mice were procured from DooYeol Biotech in Seoul, Republic of Korea. They were housed in standard laboratory conditions with ad libitum access to food and water and acclimatized for 7 d. Experimental colitis was induced with 2.5% dextran sodium sulfate (DSS; MP Biomedicals, Solon, OH, USA) in drinking water for 20 d, alternated with tap water. The procedure involved the administration of 2.5% DSS in the drinking water of mice for 6 d, followed by a 7 d period of tap water administration, then re-administration of 2.5% DSS for another 7 d.

#### 2.2.2. The Intestinal Barrier-Protective Effects of EIF on Ulcerative Colitis

Following the induction of experimental colitis, the mice were divided into four groups (n = 8): control, negative control (N.C., DSS), positive control (P.C., DSS + mesalamine), and EIF (DSS + EIF). The P.C. and EIF groups received 200 mg/kg mesalamine (Sigma-Aldrich, St. Louis, MO, USA, Cat: PHR1060) and 200 mg/kg EIF, respectively. According to the Chinese Medical Herbology and Pharmacology [29], the recommended human dose of EIF ranges from 6 to 15 g/day. Using the body surface area conversion ratio (m^2^/kg) between humans and mice [30], the equivalent mouse dose ranges from 1.2 to 3.1 g/kg body weight. Considering the 15.8% extraction efficiency of EIF, the dose equivalent to humans ranges from 194.8 to 487.1 mg/kg. Therefore, a dose of 200 mg/kg was selected for this experiment. Mesalamine (200 mg/kg), known for its anti-inflammatory effects and therapeutic efficacy in DSS-induced colitis and IBD treatment, was selected as the positive control based on previous reports [31,32,33]. The disease activity index (DAI) was assessed using the scoring system from previous studies [34,35].

After euthanasia with avertin (Sigma-Aldrich), spleen weight and large intestine length were recorded, and colonic segments were analyzed histopathologically. Severity and extent of inflammation and crypt damage were scored, and ratios of Masson’s trichrome (MT)-positive and periodic acid–Schiff (PAS)-positive areas were measured. ZO-1 and occludin antigen retrievals were performed using citrate and Tris-EDTA buffers, respectively. Primary antibodies against ZO-1 and occludin, followed by HRP Goat Anti-Rabbit IgG, were used for immunostaining, visualized with peroxidase and DAB Chromogen/Substrate (High Contrast) (ScyTek, Logan, UT, USA), and counterstained with hematoxylin. Images were acquired using the Motic digital slide assistant (Motic, Kowloon Bay, Hong Kong) and Fiji [36]. The expression levels of the target genes were measured using TaqMan probes (Table 1; Thermo Fisher Scientific Inc., Waltham, MA, USA) by real-time qPCR analysis. Detailed information on the in vivo studies is provided in the Supplementary Materials.

### 2.3. In Vitro Study

#### 2.3.1. Cell Culture

Murine RAW264.7, J774a.1, and BMDM cells were cultured in media with 10% FBS and antibiotics. The BMDMs were derived from C57BL/6 mouse bone marrow cells and cultured with macrophage colony-stimulating factor. The detailed protocol is described in the Supplementary Materials.

#### 2.3.2. Cell Viability and Toxicity of EIF

RAW264.7, J774a.1, and BMDM cells were stabilized and treated with EIF and its components (tryptanthrin, indigo, and indirubin). Cell viability was assessed using CCK-8 solution, and absorbance was measured at 450 nm with an ELISA microplate reader. The detailed protocol is described in the Supplementary Materials.

#### 2.3.3. Measurement of Nitic Oxide (NO) and Pro-Inflammatory Cytokine Levels

To measure NO production in RAW264.7 cells, 100 μL of the supernatant obtained in Section 2.3.2 was mixed with Griess reagent. NO production was quantified using a standard curve prepared with sodium nitrate (NaNO_2_). The supernatants from cultured RAW264.7 cells served as the samples, and levels of pro-inflammatory cytokines in the supernatants were quantified using an ELISA Kit (R&D Systems, Minneapolis, MN, USA) [37].

J774a.1 (1 × 10^5^ cells/200 mL/well) and BMDM cells (4 × 10^4^ cells/200 mL/well) were seeded in 96-well plates and incubated for approximately 18 h for stabilization. The cells were primed with LPS (100 ng/mL) for 6 h, followed by treatment with various concentrations of EIF and compounds for 30 min. Subsequently, 2 mM ATP was added for another 30 min, and the supernatant was collected to measure the expression of IL-1β. Cell viability was assessed using the CCK-8 assay. MCC950, an NLRP3 inflammasome inhibitor, and dexamethasone (DEX) were used as positive controls.

#### 2.3.4. NF-κB Luciferase Reporter-Based Assay

A luciferase assay was used to examine NF-κB inhibition by EIF in NF-κB Reporter (Luc)-RAW264.7 macrophages, with viability measured using CCK-8. The detailed protocol is described in the Supplementary Materials.

#### 2.3.5. Western Blotting

J774a.1 cells were lysed after incubation, and the proteins were quantified, subjected to SDS-PAGE, transferred to a membrane, and analyzed using primary antibodies for IL-1β, pro-IL-1β, NLRP3, ASC, β-actin, caspase-1, and pro-caspase-1 (Cell Signaling, Danvers, MA, USA), along with secondary antibodies diluted at 1:5000. The detailed protocol is described in the Supplementary Materials.

### 2.4. Statistical Analyses

Statistical differences among groups were analyzed using GraphPad Prism v9 software (GraphPad Software, San Diego, CA, USA). Body weight and DAI were analyzed using two-way analysis of variance (ANOVA) with Dunnett’s comparison test. Colon length was tested by a parametric unpaired two-tailed *t*-test. For the spleen index, H&E, MT, PAS, IHC, RT-qPCR, Western blotting, CCK, and ELISA results, one-way ANOVA with Dunnett’s post hoc test was used to determine statistical differences between the experimental groups. We tested for normality using the Kolmogorov–Smirnov (K-S) test and Q-Q plots to confirm whether the results followed a normal distribution.

## 3. Results

### 3.1. Effects of EIF in DSS-Induced Colitis

**Figure 1:**
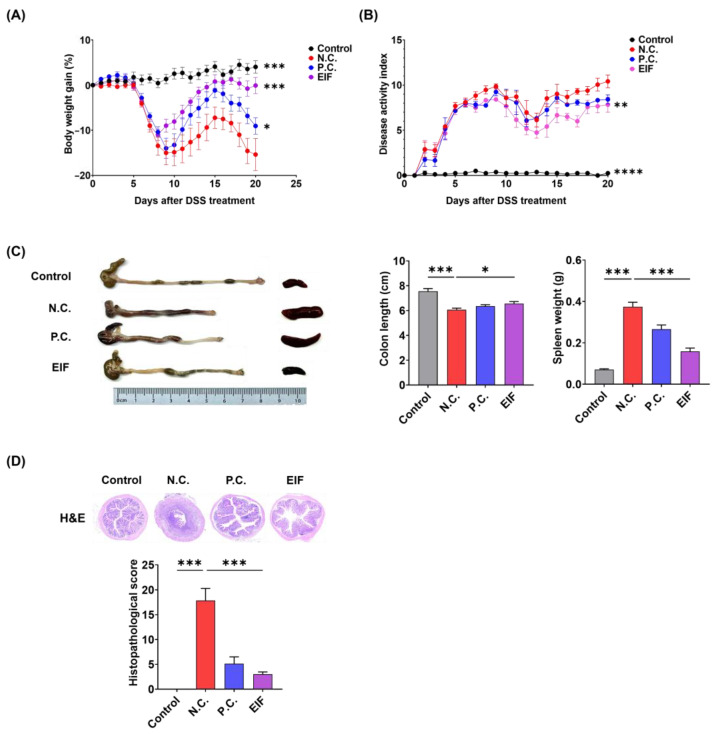
Therapeutic effects of the hydroethanolic extract of Isatidis Folium (EIF) on dextran sulfate sodium (DSS)-induced colitis in mice (n = 8). (**A**) The body weight change profile showed that EIF treatment mitigated weight loss. (**B**) The Disease Activity Index (DAI) indicated reduced colitis severity with EIF. Data are presented as means ± standard errors; ** *p* < 0.01, and **** *p* < 0.0001 versus the negative control (N.C.) group (two-way ANOVA with Dunnett’s comparison as the post hoc test). (**C**) Colon length comparison demonstrated less shortening in EIF-treated mice, and the spleen index indicated reduced spleen enlargement. Data are presented as means ± standard errors; * *p* < 0.05 and *** *p* < 0.001 versus the N.C. group (two-tailed Student’s *t*-test). (**D**) Hematoxylin and eosin (H&E)-stained colonic sections revealed reduced inflammation and tissue damage in EIF-treated mice. Data are presented as means ± standard errors; *** *p* < 0.001 versus N.C. group (one-way ANOVA with Dunnett’s comparison as the post hoc test).

In the mouse model of DSS-induced colitis, symptoms such as bloody stools, weight loss, colonic shortening, and mucosal ulcers were observed. Treatment with EIF (200 mg/kg) mitigated body weight loss compared to that in DSS-treated mice [N.C. (−15.4) vs. EIF (−0.1), *p* < 0.001]. The P.C. (DSS + mesalamine) treatment group also exhibited alleviated weight loss compared to the N.C. group, but it was not as effective as in the EIF treatment group [N.C. (−15.4) vs. P.C. (−9.0), *p* < 0.05] (Figure 1A). DAI scores, reflecting colitis severity, were reduced in the EIF-treated group compared to those in the negative control group (N.C.) by day 20 [N.C. (10.4) vs. EIF (7.8), *p* < 0.01] (Figure 1B). EIF treatment also prevented DSS-induced colon shortening [N.C. (6.1) vs. EIF (6.6), *p* < 0.05] (Figure 1C). Histological evaluation revealed that EIF treatment attenuated mucosal ulceration, crypt loss, and inflammatory cell infiltration in the colonic tissue of DSS-induced mice [N.C. (17.9) vs. EIF (3.0), *p* < 0.001] (Figure 1D).

### 3.2. Modulatory Effects of EIF on Histopathological Changes in DSS-Induced Colitis

**Figure 2:**
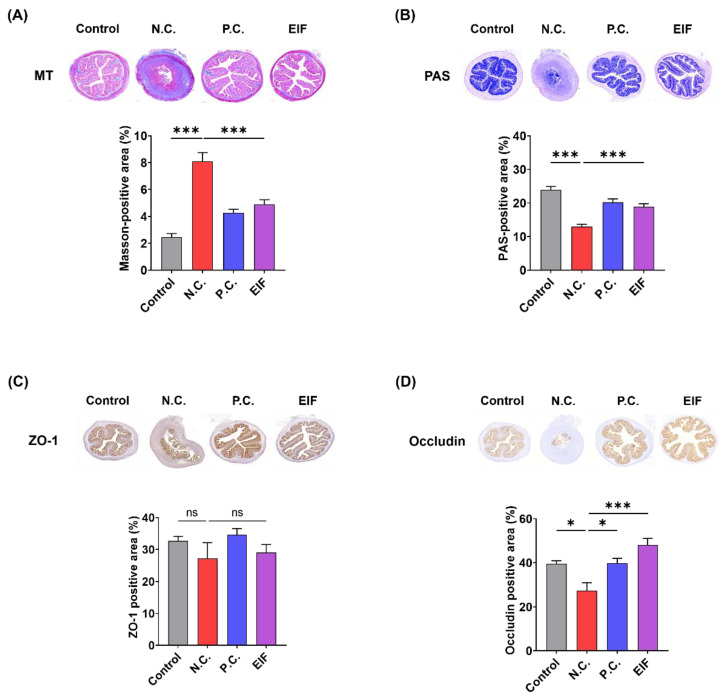
The histopathological changes in DSS-induced ulcerative colitis were evaluated. (**A**) Masson’s trichrome (MT) staining revealed collagen deposition indicative of fibrosis in colonic sections. (**B**) Periodic acid–Schiff (PAS) staining showed goblet cell preservation and mucin production. Immunohistochemistry (IHC) staining demonstrated the expression of tight junction proteins, with (**C**) ZO-1 and (**D**) occludin. The data were analyzed using one-way ANOVA with Dunnett’s post hoc test, presented as means ± standard errors. Statistical significance was marked as * *p* < 0.05, and *** *p* < 0.001 versus the N.C. group. n = 8 (**A**,**B**) and n = 6 (**C**,**D**). ns., not significant.

The histopathological changes of DSS-induced ulcerative colitis were evaluated using MT and PAS staining. The N.C. group exhibited more pronounced fibrosis and loss of goblet cells than the control group. However, in the EIF-treated group, fibrosis severity decreased [N.C. (8.1) vs. EIF (4.9), *p* < 0.001] and the staining area of goblet cells increased [N.C. (13.0) vs. EIF (18.9), *p* < 0.001] compared to that in the N.C. group (Figure 2A,B). Immunohistochemistry (IHC) analysis of ZO-1 and occludin was performed to investigate changes in tight junction protein expression. Occludin levels decreased in colon tissue of the N.C. group compared to those in the control group; however, higher expression levels were observed in the EIF-treated group than in the N.C. group [N.C. (27.3) vs. EIF (48.2), *p* < 0.001] (Figure 2D). However, no significant differences were observed in the expression of ZO-1 (Figure 2C). In addition, EIF was observed to exhibit either better or similar effects compared to P.C. (mesalamine), which is used for treatment of IBD. These findings suggest that EIF alleviates DSS-induced colitis by protecting the intestinal barrier.

### 3.3. Modulatory Effects of EIF on Inflammasome Component Levels in DSS-Induced Colitis

**Figure 3:**
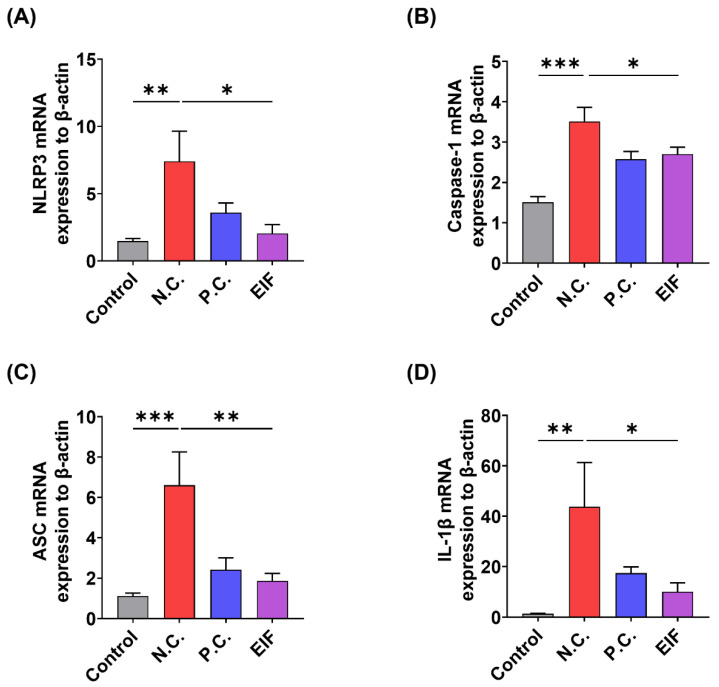
Effects of EIF on inflammasome component gene expression in DSS-induced ulcerative colitis (n = 4). RT-qPCR analysis of (**A**) NLRP3, (**B**) caspase-1, (**C**) ASC, and (**D**) IL-1β in the intestines of DSS-induced colitis mice. Data are presented as the means ± standard errors from three independent experiments. Statistical analysis was performed using one-way ANOVA followed by Dunnett’s post hoc test. Statistical significance compared to the N.C. group is indicated as * *p* < 0.05, ** *p* < 0.01, and *** *p* < 0.001.

The changes in the expression of inflammasome component genes (NLRP3, caspase-1, ASC) and IL-1β in the colon tissue of DSS-induced colitis mice following EIF treatment were examined using RT-qPCR. The results showed that compared to that in the control group, the N.C. group exhibited a significant increase in the expression of inflammasome components and IL-1β. However, the levels of NLRP3 (N.C. vs. EIF, *p* < 0.05), caspase-1 (N.C. vs. EIF, *p* < 0.05), ASC (N.C. vs. EIF, *p* < 0.01), and IL-1β (N.C. vs. EIF, *p* < 0.05) were reduced following EIF treatment (Figure 3).

### 3.4. Modulatory Effects of EIF on LPS-Induced Inflammatory Responses in RAW264.7 Cells

**Figure 4:**
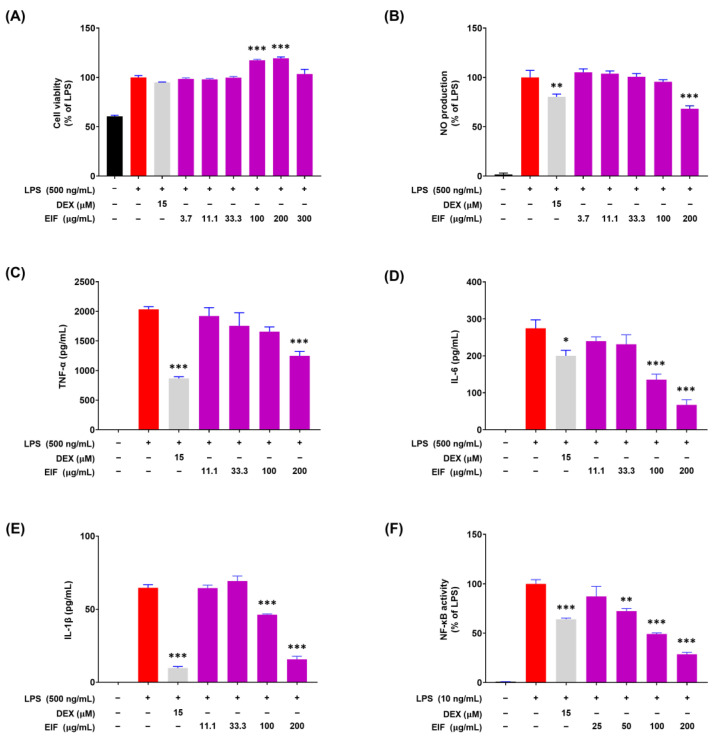
Modulatory effects of the hydroethanolic extract of Isatidis Folium (EIF) on LPS-induced inflammatory responses in RAW264.7 macrophages. (**A**) Cell viability was determined using the Cell Counting Kit-8 (CCK-8) assay, showing that EIF maintained cell viability in LPS-treated RAW264.7 cells within a non-cytotoxic range. (**B**) Nitric oxide (NO) production was measured by the Griess reagent assay. (**C**) Tumor necrosis factor-alpha (TNF-α), (**D**) interleukin-6 (IL-6), and (**E**) interleukin-1 beta (IL-1β) levels were significantly lowered by EIF treatment, assessed via ELISA. (**F**) NF-κB activity was suppressed by EIF, as indicated by the luciferase reporter assay. Dexamethasone (DEX) was used as a positive control. Data are presented as the means ± standard errors from three independent experiments. Statistical analysis was performed using one-way ANOVA followed by Dunnett’s post hoc test. Statistical significance compared to the N.C. group is indicated as * *p* < 0.05, ** *p* < 0.01, and *** *p* < 0.001. N.D., not detected.

To confirm the anti-inflammatory activity of EIF, changes in the expression of inflammatory mediators were examined in RAW264.7 macrophage cells. EIF treatment (3.7–200 μM) and LPS treatment (500 ng/mL) for 21 h did not significantly affect cell viability (Figure 4A). Within the non-cytotoxic range, EIF treatment at 200 μM resulted in a 31.7% decrease in NO production (Figure 4B). The expression of pro-inflammatory cytokines significantly increased upon LPS (500 ng/mL) treatment; however, it was reduced upon EIF treatment at 200 μM, resulting in decreases of 38.6% for TNF-α, 75.5% for IL-6, and 75.7% for IL-1β (Figure 4C–E). In addition, NF-κB activity, involved in the expression of these inflammatory mediators, decreased in a concentration-dependent manner upon EIF treatment (Figure 4F). Moreover, the results showed a similar trend compared to DEX, which was used as the positive control.

### 3.5. Effect of EIF on the Inhibition of Proteins Related to LPS/ATP-Induced Inflammasomes

**Figure 5:**
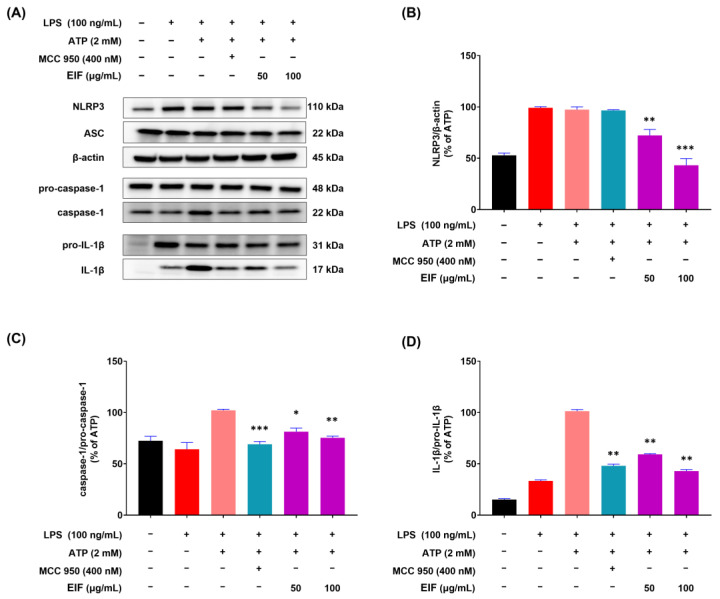
Expression of inflammasome component proteins (NLRP3, ASC, and caspase-1) and IL-1β in J774a.1 cells treated with EIF. (**A**) Western blot analysis showed levels of NLRP3, ASC, caspase-1, IL-1β, and β-actin. Quantification of protein levels revealed (**B**) reduced NLRP3, (**C**) decreased caspase-1, and (**D**) lower IL-1β expression in EIF-treated cells compared to those in the LPS/ATP-treated group. MCC950, an NLRP3 inflammasome inhibitor, was used as a positive control. Data are presented as the means ± standard errors from three independent experiments. Statistical analysis was performed using one-way ANOVA followed by Dunnett’s post hoc test. Statistical significance compared to the LPS/ATP-treated group is indicated as * *p* < 0.05, ** *p* < 0.01, and *** *p* < 0.001.

Given EIF inhibition of the inflammatory response in RAW264.7 macrophages induced by LPS, the next step was to investigate its inhibitory effects on the NLRP3 inflammasome-mediated inflammatory response. The model comprised J774a.1 macrophages stimulated with LPS and ATP to induce inflammasome formation, and Western blot results demonstrated a significant reduction in the protein expression levels of NLRP3 (*p* < 0.001), caspase-1 (*p* < 0.01), and IL-1β (*p* < 0.01) by EIF at 100 μg/mL (Figure 5). In addition, the inflammasome inhibitor MCC950 suppressed the expression of caspase-1 (*p* < 0.001) and IL-1β (*p* < 0.01) similarly to EIF but did not affect the expression of NLRP3.

### 3.6. Effect of EIF Contituents on J774a.1 and BMDM Cells Induced by LPS/ATP

**Figure 6:**
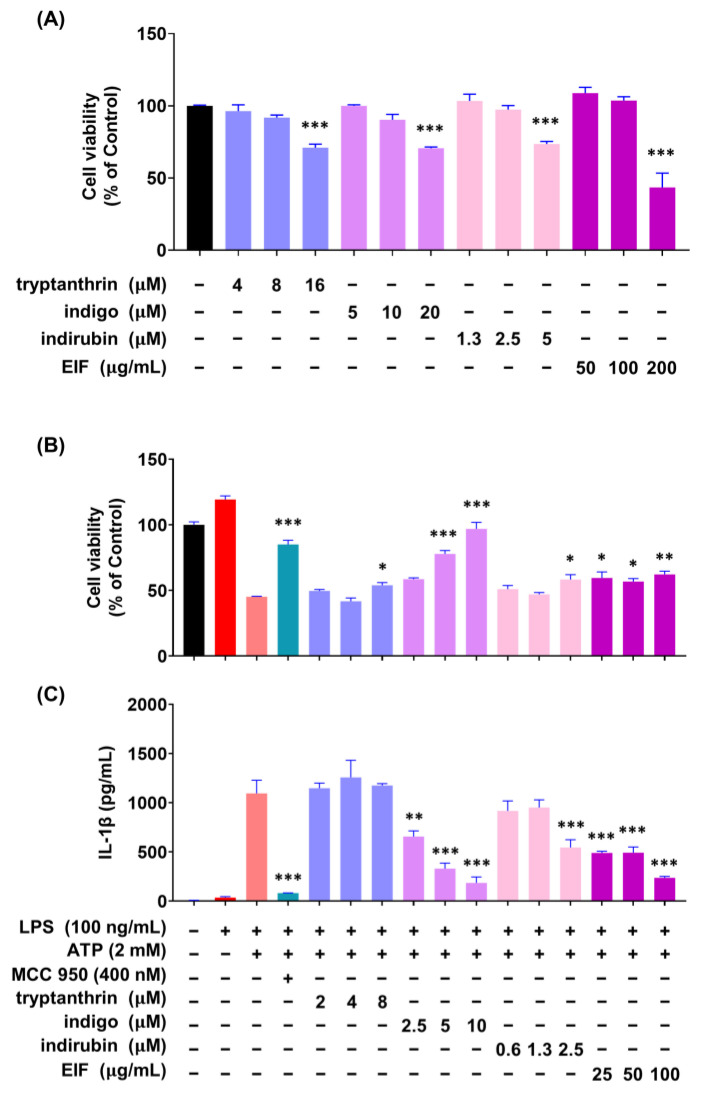
Effect of EIF constituents on J774a.1 macrophages. (**A**) Cell viability was assessed in cells treated only with EIF and its components. In LPS/ATP-treated cells, EIF constituents were tested for their effect on (**B**) cell viability and (**C**) IL-1β expression. MCC950, an NLRP3 inflammasome inhibitor, was used as the positive control. Data are presented as the means ± standard errors from three independent experiments. Statistical analysis was performed using one-way ANOVA followed by Dunnett’s post hoc test. Statistical significance compared to the LPS/ATP-treated group is indicated as * *p* < 0.05, ** *p* < 0.01, and *** *p* < 0.001.

**Figure 7:**
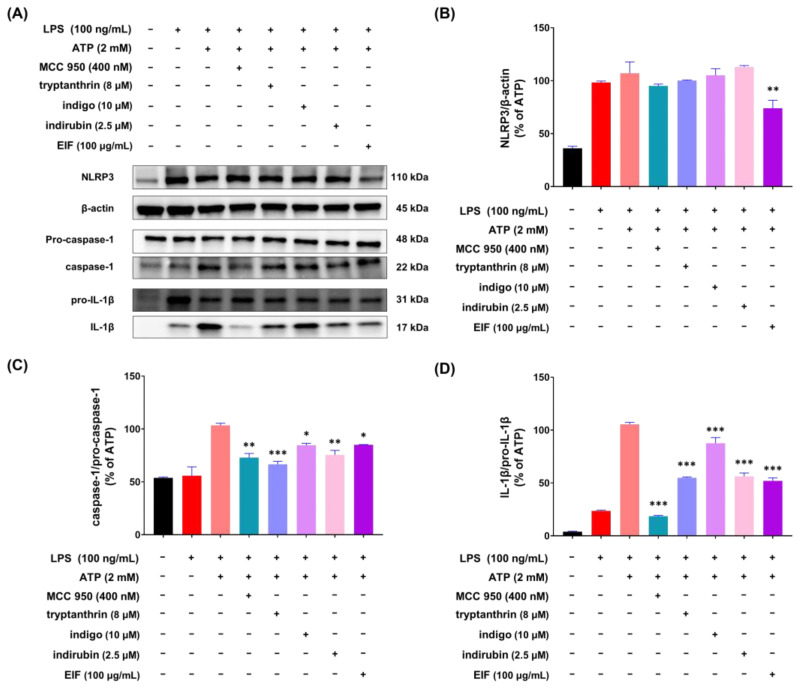
Expression of inflammasome component proteins and IL-1β in J774a.1 macrophage cells treated with EIF constituents. In LPS/ATP-treated J774a.1 cells, EIF constituents were tested for their effect on protein expression. (**A**) Western blot analysis and protein levels of (**B**) NLRP3, (**C**) caspase-1, and (**D**) IL-1β. MCC950, an NLRP3 inflammasome inhibitor, was used as a positive control. Data are presented as the means ± standard errors from three independent experiments. Statistical analysis was performed using one-way ANOVA followed by Dunnett’s post hoc test. Statistical significance compared to the LPS/ATP-treated group is indicated as * *p* < 0.05, ** *p* < 0.01, and *** *p* < 0.001.

**Figure 8:**
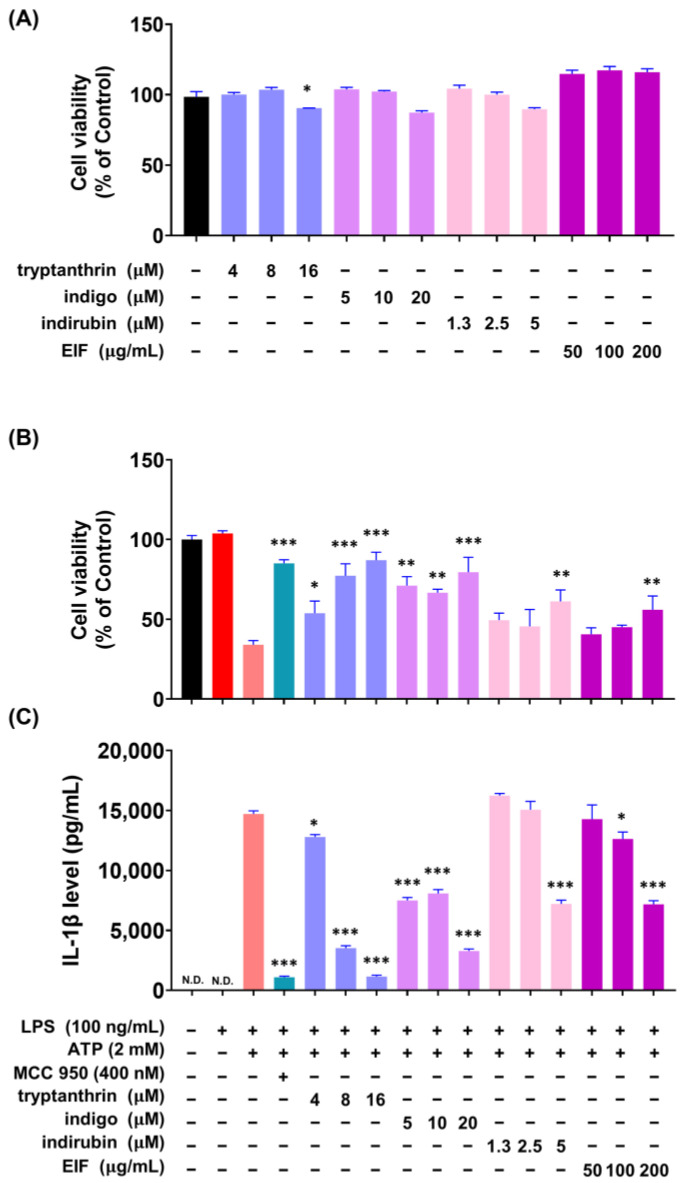
Effect of EIF constituents on bone marrow-derived macrophages (BMDMs). (**A**) Cell viability was assessed in cells treated only with EIF and its components. In LPS/ATP-treated BMDMs, EIF constituents were tested for their effects on (**B**) cell viability and (**C**) IL-1β expression. MCC950, an NLRP3 inflammasome inhibitor, was used as a positive control. Data are presented as the means ± standard errors from three independent experiments. Statistical analysis was performed using one-way ANOVA followed by Dunnett’s post hoc test. Statistical significance compared to the LPS/ATP-treated group is indicated as * *p* < 0.05, ** *p* < 0.01, and *** *p* < 0.001. N.D., not detected.

To investigate whether the constituents of EIF influence its NLRP3 inflammasome-mediated inflammatory suppression activity, we confirmed the presence of well-known constituents of EIF, tryptanthrin, indigo, and indirubin, using UPLC-MS/MS. The results demonstrated the presence of all three constituents (Figure S1). To assess the impact of EIF and its constituents on the viability of LPS/ATP-induced J774a.1 and BMDM cells, cell viability was measured using CCK-8 analysis. In J774a.1 cells, treatment with LPS and ATP significantly suppressed cell viability compared to that in the control group, whereas pretreatment with EIF and its constituents, except for tryptanthrin, improved viability (LPS/ATP vs. indigo, indirubin, and EIF; *p* < 0.001, *p* < 0.05, and *p* < 0.01, respectively, at the highest concentration) (Figure 6B). Similarly, in BMDM cells, treatment with LPS and ATP decreased cell viability; however, pretreatment with EIF and its constituents enhanced viability (LPS/ATP vs. tryptanthrin, indigo, indirubin, and EIF; *p* < 0.001, *p* < 0.001, *p* < 0.01, and *p* < 0.01, respectively, at the highest concentration) (Figure 7B). The expression of IL-1β in both J774a.1 and BMDM cells increased after ATP treatment and decreased proportionally to the improvement in cell viability observed with each sample (Figure 6C and Figure 8C). Western blot results showed that EIF and its constituents inhibited the protein expression levels of caspase-1 and IL-1β compared to those in the LPS/ATP-stimulated group. However, they did not affect NLRP3 expression, except for the EIF treatment (Figure 7).

## 4. Discussion

The DSS model is a well-established experimental model that closely resembles the early-stage symptoms of human IBD, such as weight loss, diarrhea, bloody stools, mucosal ulcers, colon atrophy, and damage to colonic epithelial cells [38,39]. This model is invaluable for understanding the pathophysiology of IBD and testing potential therapeutic interventions. The integrity of the intestinal barrier, maintained by intestinal epithelial cells, is crucial for separating the intestinal lumen from the internal environment. This barrier plays a pivotal role in protecting intestinal tissue from harmful microbes, toxins, and other damaging substances while also ensuring proper immune function [40,41,42,43]. Disruption of this barrier is a hallmark of IBD and contributes significantly to disease progression. Alternating between DSS water and tap water is a common protocol to induce cyclic inflammation. The rationale for alternating between DSS and tap water is to model relapsing-remitting colitis, which mimics flare-ups and remission observed in human IBD. Therefore, we conducted animal experiments by referring to the DSS-induced colitis mouse model from previous studies [44,45].

The findings of our study demonstrate the efficacy of EIF in significantly suppressing colitis symptoms induced by DSS. Histological analyses, including MT staining, PAS staining, and IHC assays, provided detailed insights into the protective effects of EIF. These analyses revealed that EIF effectively suppressed intestinal fibrosis, a severe complication that can lead to long-term damage in chronic colitis. Furthermore, EIF protected goblet cells, which are essential for the production and maintenance of mucin, a critical component of the mucus layer that shields the gut lining from pathogens and physical damage. EIF also inhibited the degradation of occludin protein, a key component of tight junctions between epithelial cells, which is vital for maintaining the barrier’s integrity and permeability. These findings suggest that EIF plays a crucial role in suppressing DSS-induced colitis by protecting the intestine from damage.

To further explore the anti-inflammatory properties of EIF, we investigated its effects on macrophages stimulated by LPS. When macrophages are stimulated by LPS, inflammation-related signaling pathways such as NF-κB are activated, leading to the secretion of large amounts of inflammatory mediators, including NO and pro-inflammatory cytokines, such as TNF-α, IL-6, and IL-1β [46]. Although inflammation is necessary for immune responses to eliminate antigens and inhibit infections, excessive activation can cause tissue damage and contribute to disease development [47]. Therefore, blocking the production pathways of these inflammatory mediators to reduce their secretion is crucial for improving and treating inflammation-related diseases [48]. LPS-stimulated RAW264.7 cells showed increased production of NO and pro-inflammatory cytokines, which were reduced by EIF treatment within a non-cytotoxic range. In addition, EIF inhibited NF-κB activation, indicating its involvement in inflammatory responses and reducing pro-inflammatory cytokine expression.

Given the central role of the NLRP3 inflammasome in inflammatory responses, we further investigated the effects of EIF on the NLRP3 inflammasome-mediated inflammatory response. The NLRP3 inflammasome is composed of three molecules: NLRP3 as a sensor, ASC acting as an adapter, and caspase-1 with enzymatic activity, forming a protein complex of approximately 700 kDa. Upon activation, pro-caspase-1 is converted to its active form, caspase-1, which cleaves pro-IL-1β into IL-1β for secretion outside the cell. In other words, the secretion of IL-1β due to NLRP3 inflammasome activation depends on the sequential assembly and activation of NLRP3, ASC, and caspase-1 enzymes [49]. RT-qPCR analysis results showed that EIF treatment significantly reduced the expression of inflammasome component genes (NLRP3, caspase-1, and ASC) and IL-1β in the colon tissue of DSS-induced colitis mice (Figure 3). Additionally, Western blot analysis in J774a.1 macrophages showed that EIF treatment reduced the expression of inflammasome component proteins and decreased the levels of active caspase-1 and IL-1β. However, EIF did not affect the expression of ASC, suggesting that its inhibitory effect might occur through the modulation of other components or steps in the inflammasome (Figure 5).

EIF contains well-known constituents, such as tryptanthrin, indigo, and indirubin, which have documented anti-inflammatory properties. Tryptanthrin suppresses inflammasome activity by interfering with the interaction between ASC and NLRP3/NLRC4/AIM2 [50]. In addition, indigo and indirubin inhibit the NF-κB pathway, reducing the expression of inflammatory cytokines [51,52]. To model whether IL-1β production is induced by LPS and ATP stimulation, we used both J774a.1 and BMDM cells and tested the activity of EIF and its constituents. Treatment with the constituents improved cell viability, similar to the outcome of the EIF treatment, which had been reduced by ATP treatment. In addition, the expression of IL-1β increased owing to cytotoxicity induced post-ATP treatment and decreased proportionally with enhanced viability. These findings suggest that EIF and its constituents may have an inhibitory effect on the inflammasome-mediated inflammatory response. Moreover, Western blot results showed that treatment with EIF and its constituents inhibited the protein expression levels of caspase-1 and IL-1β in LPS/ATP-stimulated J774a.1 macrophages. However, an interesting observation was that only EIF affected NLRP3 expression. This suggests a unique or synergistic effect of the whole extract, highlighting the complexity of its anti-inflammatory properties. Further research is needed to understand these mechanisms.

This study provides evidence that EIF has therapeutic potential for treating inflammation-related diseases, particularly IBD. EIF has been shown to manage inflammation by protecting the intestine from damage, inhibiting key inflammatory signaling pathways, and modulating inflammasome-mediated responses. Additionally, specific constituents of EIF, such as tryptanthrin, indigo, and indirubin, contribute to its overall anti-inflammatory effects, offering promising avenues for further research and development.

## 5. Conclusions

This study evaluated the effects of EIF on the alleviation of colitis and anti-inflammatory effects related to inflammasomes. Animal studies showed the efficacy of EIF in alleviating DSS-induced colitis by protecting against intestinal damage. Further studies revealed that EIF exhibited anti-inflammatory effects by inhibiting NF-κB activity and inflammasome activation. These results suggest EIF as a potential agent for preventing and treating inflammatory-related diseases. However, to demonstrate that EIF has benefits in maintaining barrier permeability as a potential treatment strategy for IBD, further experiments will be needed using physiological markers to assess inflammatory cell expression (MPO, F4/80) and changes in the protein and mRNA expression of markers such as Zo-1, occludin, trefoil factor 3, mucin 2, Kruppel-like factor 4, matrix metallopeptidase, and collagen type III alpha 1 in colon tissue.

## Figures and Tables

**Table 1:** Information on quantitative reverse-transcription polymerase chain reaction (RT-qPCR).

Gene Name	Assay ID	NCBI Reference Sequence
NOD-like receptor protein 3 (NLRP3)	Mm00840904_m1	NG_007509.2
Caspase-1 Apoptosis-associated speck-like protein (ASC)	Mm00438023_m1 Mm00445747_g1	NM_001043585.1 NG_029446.1
β-actin	Mm00607939_s1	NM_007393.5

## Data Availability

The original contributions presented in the study are included in the article and Supplementary Materials, further inquiries can be directed to the corresponding author.

## Supplementary Materials

The following supporting information can be downloaded at: https://www.mdpi.com/article/10.3390/nu16193323/s1, Figure S1: UPLC-MS/MS analysis of EIF; Table S1: UPLC-MS/MS analysis of EIF. Reference [28,53] is cited in Supplementary Materials.
