# Supplementary material for: Isatidis Folium Represses Dextran Sulfate Sodium-Induced Colitis and Suppresses the Inflammatory Response by Inhibiting Inflammasome Activation
Source: Nutrients. 2024 Sep 30;16(19):3323. doi: 10.3390/nu16193323 (PMC11478736; doi:10.3390/nu16193323)
Supplement: Supplementary file 1 [file nutrients-16-03323-s001.zip › nutrients-3207303-supplementary.pdf]

Supplementary File

Isatidis Folium Represses Dextran Sulfate Sodium-Induced Colitis and Suppresses the Inflammatory Response by Inhibiting Inflammasome Activation

You Chul Chung[1,†], Ami Lee[1,2,†], Chan Ho Jang[1], Jin Ah Ryuk[1], Hyunil Ha[1] and Youn-Hwan Hwang[1,2*]

[1] KM Convergence Research Division, Korea Institution of Oriental Medicine, 1672 Yuseong-daero, Yuseong-gu, Deajeon 34054, Republic of Korea; jyc8385@kiom.re.kr (Y.C.C.); dmb01367@kiom.re.kr (A.L.); chjang78@kiom.re.kr (C.H.J.); yukjinah@kiom.re.kr (J.A.R.); hyunil74@kiom.re.kr (H.H.)

[2] Korean Convergence Medical Science Major, KIOM School, University of Science & Technology (UST), Deajeon 34054, Republic of Korea

* Correspondence: hyhhwang@kiom.re.kr; Tel.: +82-42-868-9260

[†] These authors contributed equally to this work.

This file includes:

1. **Supplementary Material and Methods**

2. **Supplementary Table S1**
   UPLC-MS/MS analysis of EIF

3. **Supplementary Figure S1**
   UPLC-MS/MS analysis of EIF

1. Supplementary Materials and Methods

Animal studies

Animal group and administrations

To evaluate the pharmacological effects of EIF on ulcerative colitis, animals were randomly allocated into four groups (n = 8): control, negative control (N.C., DSS), positive control (P.C., DSS + mesalamine), and EIF (DSS + EIF). Colitis was induced by adding 2.5% (w/v) DSS to their drinking water for 20 d. The procedure involved the administration of 2.5% DSS in the drinking water of mice for 6 d, followed by 7 d period of tap water, and re-administration of 2.5% DSS for another 7 d. Throughout the study period, the control and N.C. groups received only water, whereas the P.C. and EIF groups were treated with 200 mg/kg mesalamine (Sigma-Aldrich, Cat: PHR1060) and 200 mg/kg EIF, respectively.

Colitis severity evaluation and histopathological analysis

DAI was calculated daily after the start of DSS treatment by summing scores for weight loss percentage (none, 0; 1–5%, 1; 5–10%, 2; 10–15%, 3; >15%, 4), stool consistency (normal, 0; loose stools, 2; watery diarrhea, 4), and rectal bleeding (no bleeding, 0; slight bleeding, 2; gross bleeding, 4). At the end of the experiment, mice were humanely euthanized using avertin (Sigma-Aldrich). Thereafter, spleen weight and large intestine length were measured. Colonic segments were fixed in 10% neutral-buffered formalin for histopathological assessment. Paraffin-embedded colon

samples were sectioned and stained with hematoxylin and eosin (H&E), Masson's trichrome (MT), and periodic acid-Schiff (PAS). Inflammation severity and extent were evaluated using a scoring system ranging from 0 to 3, whereas crypt damage was scored from 0 to 4. The total histopathological score was calculated by multiplying the score for each feature (severity of inflammation, extent of inflammation, and crypt damage) by the score representing the percentage of involvement. In MT and PAS-stained images, the ratio of the Masson-positive area (i.e., the area stained blue by MT) or the PAS-positive area (i.e., the area stained blue by PAS stain, such as goblet cells) in the entire colon tissue area of each sample was measured and expressed as a percentage.

Immunohistochemistry (IHC)

Antigen retrieval for ZO-1 involved boiling in citrate buffer (pH 6.0), whereas for occludin, it was achieved in Tris-EDTA buffer (pH 9.0). Following blocking, sections were exposed to primary antibodies against ZO-1 (diluted 1:250, Abcam, San Francisco, CA, USA) and occludin (diluted 1:200, Abcam) under suitable conditions. Subsequently, an immune response was stimulated using the ImmPRESS® HRP Goat Anti-Rabbit IgG Polymer Detection Kit (Vector Laboratories, Burlingame, CA, USA). Visualization was facilitated using peroxidase (Vector Laboratories) and the DAB Chromogen/Substrate Kit (High Contrast) (ScyTek, Logan, UT, USA), accompanied by counterstaining using hematoxylin. Image scanning, analysis, and editing adhered to

protocols detailed for MT and PAS.

RT-qPCR analysis

The total RNA of colon tissue was extracted using the RNeasy Plus Mini Kit (Qiagen, Hilden, Germany). Equal amounts of RNA (1000 ng) were quantified, and cDNA reverse transcription was performed using a cDNA synthesis kit (High-Capacity cDNA Reverse Transcription Kit; Thermo Fisher Scientific Inc.). RT-qPCR was performed using Taqman Gene Expression Master Mix (Thermo Fisher Scientific Inc.).

In vitro study

Cell culture

Murine macrophage RAW264.7 and J774a.1 were obtained from the Korean Cell Line Bank (Jongno-gu, Seoul, Korea) and maintained in Dulbecco's modified Eagle's medium (DMEM; ThermoFisher, MA, USA) containing 10% fetal bovine serum (FBS; ThermoFisher) and 1% penicillin/streptomycin at 37 °C in 5% CO_2 atmosphere (humidified). Mouse primary bone-marrow-derived macrophages (BMDMs) were differentiated from bone marrow cells isolated from the femurs and tibias of C57BL/6 mice. The bone marrow cells were cultured for 6 d in Iscove's modified Dulbecco's medium containing 60 ng/mL macrophage colony-stimulating factor (M-CSF), 10% FBS, 1% penicillin/streptomycin, 1× Minimum Essential Medium (Gibco 11140-050,

Carlsbad, CA, USA), and 1 mM sodium pyruvate (Gibco, 11360-070). Thereafter, the differentiated cells were used for experiments.

Cell viability and toxicity of EIF

RAW264.7 cells (4×10^4 cells/well) were stabilized for 24 h in a 37 °C, 5% CO_2 incubator, and subsequently treated with various concentrations of EIF. After a 3 h pretreatment, LPS (*E. coli* O110, Sigma-Aldrich Co., St. Louis, MO, USA) was added to the cells at a concentration of 500 ng/mL and incubated for 21 h. J774a.1 and BMDM cells (4×10^4 cells/well) were also stabilized for 24 h under the same conditions, and subsequently treated with various concentrations of EIF and its ingredient compounds (tryptanthrin, indigo, and indirubin) for 24 h. After collecting the culture supernatant, 100 μL of DMEM containing 10% Cell Counting Kit-8 solution (CCK-8, Dojindo Molecular Technologies, Inc., USA) was added to each well to assess cell viability. Subsequently, absorbance was measured at 450 nm using an ELISA microplate reader (BioTek Instruments, Winooski, VT, USA).

NF-κB luciferase reporter-based assay

A luciferase assay was used to examine the inhibition of NF-κB luciferase reporter gene expression by EIF in LPS-stimulated NF-κB Reporter (Luc)-RAW264.7 macrophages. The cells were seeded in a 96-well plate at a density of 4×10^4 cells/well and incubated for approximately 21 h for stabilization. Following treatment with EIF

for 21 h, cells were treated with 10 ng/mL LPS for 3 h. Cell viability was evaluated using the CCK-8 assay. Reporter quantitation in cell lysates used the Luciferase Assay System (Promega, Madison, USA) and a TriStar LB941 Luminometer (Berthold technologies, Germany) according to the manufacturer's instructions.

Western blotting

J774a.1 cells (1×10^6 cells/well) were seeded in 6-well plates and cultured under the same conditions as in other experiments. After washing twice with cold phosphate-buffered saline, cells were lysed with M-PERTM lysis buffer (Thermo Fisher Scientific) containing 1% protease and a phosphatase inhibitor cocktail (Bio-Rad, Hercules, CA, USA). The extracted cellular proteins were quantified using the BCA protein assay kit (Thermo Fisher Scientific), and 20 μg of protein was subjected to electrophoresis on a 12% sodium dodecyl sulfate-polyacrylamide gel. Separated proteins were transferred to a polyvinylidene difluoride membrane (Bio-Rad) for 2 h. After blocking with 5% skim milk for 1 h, the membrane was incubated with primary antibodies against NLRP3, ASC, pro-caspase-1, caspase-1, pro-IL-1β, IL-1β, and β-actin (rabbit monoclonal antibodies, Cell Signaling, Danvers, MA, USA) diluted 1:1,000. Following incubation with secondary antibodies (HRP-conjugated anti-rabbit IgG diluted 1:5,000) for 2 h, the membrane was washed six times for 10 min each with TBST (TBS containing 0.05% Tween-20). Thereafter, protein bands were visualized using an Enhanced Chemiluminescence Kit (Bio-Rad) and analyzed.

Ultra-high performance liquid chromatography-tandem mass spectroscopy (UPLC-MS/MS)

To analyze the EIF, a Dionex UltiMate 3000 UPLC system equipped with a Thermo Q-Exactive mass spectrometer was used. Chromatographic separation was performed using an Acquity BEH C18 column (100 × 2.1 mm,1.7 μm) and the gradient setting was performed as described by Shim et al., using 0.1% formic acid in water and acetonitrile [1]. The identification of phytochemicals (tryptanthrin, indigo, and indirubin) in EIF was performed by comparing their retention times and mass spectral data (Table S1) with reference standards or based on a previous report [2].

Table S1. Characterization of identified compounds of EIF by UPLC-MS/MS.

No.	Theoretical (m/z)	Measured (m/z)	Error (ppm)	Adduct	R_t (min)	Formula	Fragments (m/z)	Identifications
1	249.0659	249.0661	1.0081	$[M+H]^+$	12.93	$C_{15}H_8N_2O_2$	130	Tryptanthrin [2]
2	263.0815	263.0818	0.9753	$[M+H]^+$	14.57	$C_{16}H_{10}N_2O_2$	235, 219	Indigo*
3	263.0815	263.0817	0.8593	$[M+H]^+$	15.65	$C_{16}H_{10}N_2O_2$	235	Indirubin*

R_t, Retention time; *, Compared with the R_t and mass spectra of genuine reference compounds.

3. Peak chromatogram

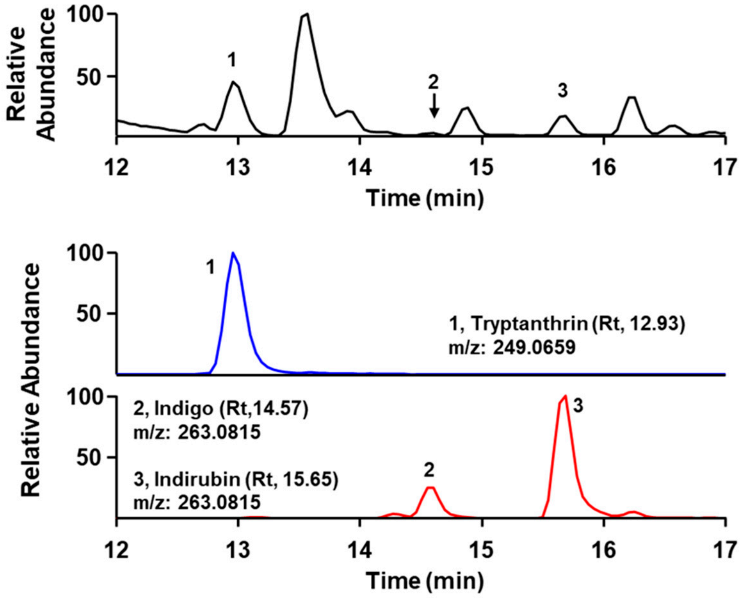

Figure S1. The base peak chromatogram of EIF in positive ionization mode using UPLC-MS/MS (upper panel), and the extracted ion chromatogram for the identified compounds tryptanthrin, indigo, and indirubin with retention times (lower panel) between 12 and 17 min.

References

1. Shim, K.S.; Gu, D.R.; Hwang, Y.H.; Yang, H.; Ryuk, J.A.; Ha, H. Water extract of Fritillariae thunbergii Bulbus inhibits RANKL-mediated osteoclastogenesis and ovariectomy-induced trabecular bone loss, *Molecules* **2021**, 27, doi.org/10.3390/molecules27010169.
2. Liau, B.C.; Jong, T.T.; Lee, M.R.; Chen, S.S. LC-APCI-MS method for detection and analysis of tryptanthrin, indigo, and indirubin in daqingye and banlangen. *J. Pharm. Biomed. Anal*. **2007**, *43*, 346-351, doi.org/10.1016/j.jpba.2006.06.029.
